# Glutamate acid decarboxylase 1 promotes metastasis of human oral cancer by β-catenin translocation and MMP7 activation

**DOI:** 10.1186/1471-2407-13-555

**Published:** 2013-11-21

**Authors:** Ryota Kimura, Atsushi Kasamatsu, Tomoyoshi Koyama, Chonji Fukumoto, Yukinao Kouzu, Morihiro Higo, Yosuke Endo-Sakamoto, Katsunori Ogawara, Masashi Shiiba, Hideki Tanzawa, Katsuhiro Uzawa

**Affiliations:** 1 Department of Clinical Molecular Biology, Graduate School of Medicine, Chiba University, 1-8-1 Inohana, Chuo-ku, Chiba 260-8670, Japan; 2 Department of Dentistry and Oral-Maxillofacial Surgery, Chiba University Hospital, 1-8-1 Inohana, Chuo-ku, Chiba 260-8670, Japan; 3 Department of Medical Oncology, Graduate School of Medicine, Chiba University, 1-8-1 Inohana, Chuo-ku, Chiba 260-8670, Japan

**Keywords:** Glutamate acid decarboxylase 1, β-catenin, Matrix metalloproteinase-7, 3-mercaptopropionic acid, Metastasis, Oral squamous cell carcinoma

## Abstract

**Background:**

Glutamate decarboxylase 1 (GAD1), a rate-limiting enzyme in the production of γ-aminobutyric acid (GABA), is found in the GABAergic neurons of the central nervous system. Little is known about the relevance of GAD1 to oral squamous cell carcinoma (OSCC). We investigated the expression status of GAD1 and its functional mechanisms in OSCCs.

**Methods:**

We evaluated GAD1 mRNA and protein expressions in OSCC-derived cells using real-time quantitative reverse transcriptase-polymerase chain reaction (qRT-PCR) and immunoblotting analyses. To assess the critical functions of GAD1, i.e., cellular proliferation, invasiveness, and migration, OSCC-derived cells were treated with the shRNA and specific GAD1 inhibitor, 3-mercaptopropionic acid (3-MPA). GAD1 expression in 80 patients with primary OSCCs was analyzed and compared to the clinicopathological behaviors of OSCC.

**Results:**

qRT-PCR and immunoblotting analyses detected frequent up-regulation of GAD1 in OSCC-derived cells compared to human normal oral keratinocytes. Suppression of nuclear localization of β-catenin and MMP7 secretion was observed in GAD1 knockdown and 3-MPA-treated cells. We also found low cellular invasiveness and migratory abilities in GAD1 knockdown and 3-MPA-treated cells. In the clinical samples, GAD1 expression in the primary OSCCs was significantly (*P* < 0.05) higher than in normal counterparts and was correlated significantly (*P* < 0.05) with regional lymph node metastasis.

**Conclusions:**

Our data showed that up-regulation of GAD1 was a characteristic event in OSCCs and that GAD1 was correlated with cellular invasiveness and migration by regulating β-catenin translocation and MMP7 activation. GAD1 might play an important role in controlling tumoral invasiveness and metastasis in oral cancer.

## Background

Glutamate decarboxylase 1 (GAD1) catalyzes production of γ-aminobutyric acid (GABA) from L-glutamic acid, the principal inhibitory neurotransmitter in the brain [1,2]. GAD1 is associated with development of insulin-dependent diabetes mellitus [3] and many cases of the Stiff-Person syndrome [4]. The murine model of cleft palate also lacks GAD1 expression [5].

GAD1 is related closely to β-catenin expression by microarray analysis in ovarian endometrioid adenocarcinoma and Wilms’ tumor [6,7], whereas the functional interaction between GAD1 and β-catenin has not been demonstrated clearly. β-catenin is an essential component of both intercellular junctions and the canonical Wnt signaling pathway and connects the adherens junction complex with the actin cytoskeleton that binds directly to the intracellular domain of E-cadherin [8]. Disruption of β-catenin plays critical roles in the regulation of cellular invasiveness, proliferation, and migration [9-11].

The Wnt/β-catenin pathway is activated when the Wnt ligand binds to cell-surface receptors, the Frizzled family receptor, and the LRP 5/6 co-receptor. Activation of this pathway leads to inhibition of a complex comprising adenomatous polyposis coli, axis inhibition protein, and glycogen synthase kinase 3β. This complex has functions is involved in phosphorylation and degradation of β-catenin by the ubiquitin/proteosome system. After activation of the Wnt/β-catenin pathway, β-catenin translocates to the nucleus for binding to T-cell factor/lymphoid enhancer factor (TCF/LEF) and activates transcription of Wnt-targeting genes [12].

MMP7 is a Wnt-targeting gene that has been detected in several cancers, such as prostate, colon, stomach, lung, and breast [13-17] and degrades components of the extracellular matrix (ECM), including collagens (I, III, IV, and V), fibronectin, vitronectin, laminin, and elastin [18]. In the oral region, Chuang et al. demonstrated that MMP7 is closely related to invasion in OSCCs of buccal mucosa [19]. Therefore, MMP7 contributes significantly to the cellular invasiveness and metastasis of tumors.

The current study found that GAD1 is overexpressed frequently in OSCC-derived cell lines, and that GAD1 knockdown affects cellular invasiveness and migration. Based on this evidence, we proposed that GAD1 might be a therapeutic target to prevent metastasis in OSCCs.

## Methods

### Ethics statement

The Ethics Committee of the Graduate School of Medicine, Chiba University (approval number, 236) approved the study protocol, which was performed according to the tenets of the Declaration of Helsinki. All patients provided written informed consent.

### OSCC-derived cell lines and tissue samples

RIKEN BRC (Ibaraki, Japan) provided the Sa3, HO-1-u-1, KOSC-2, Ca9-22, HO-1-N-1, HSC-2, and HSC-3 cell lines through the National Bio-Resource Project of the MEXT, Tokyo, Japan. Short tandem repeat profiles confirmed the cellular identity. Primary cultured human normal oral keratinocytes (HNOKs) were used as normal controls [20,21]. All cells were grown in Dulbecco’s modified Eagle’s medium (DMEM) (Sigma, St. Louis, MO) supplemented with 10% fetal bovine serum (FBS) (Sigma) and 50 units/ml of penicillin and streptomycin (Sigma). Primary OSCCs and patient-matched normal oral epithelial samples were obtained during surgical resections of the tumors with simultaneous neck dissection at Chiba University Hospital. The average age of the patients was 64.6 years (range, 27–90 years). The mean follow-up time for all of the patients was 68.5 months (range, 24–98 months). The resected tissues were fixed in 10% buffered formaldehyde solution for pathological diagnosis and immunohistochemistry (IHC). Histopathological diagnosis of each tissue was performed according to the tumor-node-metastases classification of the International Union against Cancer.

### Preparation of cDNA

Total RNA was isolated using TRIzol Reagent (Invitrogen, Carlsbad, CA). cDNA was generated from 5 μg of total RNA using Ready-To-Go You-Prime First-Strand Beads (GE Healthcare, Buckinghamshire, UK) and oligo (dT) primer (Sigma Genosys, Ishikari, Japan).

### mRNA expression analysis

Real-time quantitative reverse transcriptase-polymerase chain reaction (qRT-PCR) was performed using a Light Cycler 480 apparatus (Roche Diagnostics GmbH, Mannheim, Germany) to evaluate the expression levels of *GAD1* mRNA in the seven OSCC-derived cell lines (HSC-2, HSC-3, Sa3, HO-1-u-1, HO-1-N-1, KOSC-2, and Ca9-22) and HNOKs. Primers were designed using the Probe Finder qRT-PCR assay design software (available at http://www.universalprobelibrary.com). The sequences of the gene-specific primers and universal probes were as follows: *GAD1* forward, 5′-CCA TGG TCG TAC CTG ACT CC-3′ and reverse, 5′-CCT GGA ACT GGC TGA ATA CC-3′ (probe #78); *MMP7* forward, 5′-TCT CCT CCG AGA CCT GTC C-3′ and reverse, 5′-GCT GAC ATC ATG ATT GGC TTT-3′ (probe #72); and *β-catenin* forward, 5′-GCT TTC AGT TGA GCT GAC CA-3′ and reverse, 5′-CAA GTC CAA GAT CAG CAG TCT C-3′ (probe #21). The PCR reactions were carried out in a final volume of 20 μl of a reaction mixture comprised of 10 μl of Light Cycler 480 Probes Master (Roche), 0.2 μl of universal probe (Roche), and 4 μM of the primers. The reaction mixture was loaded onto the PCR plate and subjected to an initial denaturation at 95°C (10 min), followed by 45 rounds of amplification at 95°C (10 sec) for denaturation, 60°C (30 sec) for annealing, and 72°C (1 sec) for extension, followed by a cooling step at 50°C for 30 seconds. The transcript amounts for the *GAD1* and other genes were estimated from the respective standard curves and normalized to glyceraldehyde-3-phosphate dehydrogenase (*GAPDH*) forward, 5′-AGCCACATCGCTCAGACAC-3′ and reverse, 5′-GCCCAATACGACCAAATCC-3′ (probe #60) transcript amounts determined in corresponding samples.

### Protein extraction

The cells were washed twice with cold phosphate buffered saline (PBS) and centrifuged briefly. The cell pellets were incubated at 4°C for 30 min in a lysis buffer (7 M urea, 2 M thiourea, 4% w/v CHAPS, and 10 mM Tris pH 7.4) with a proteinase inhibitor cocktail (Roche) to extract whole cell lysates. The protein concentrations of whole cell lysates were measured using the Bradford reagent (Bio-Rad, Richmond, CA). Cytoplasmic and nuclear fractions from cultured cells were isolated using the NE-PER Nuclear and Cytoplasmic Extraction Reagents (Thermo, Rockford, IL). The protein concentrations were measured using the BCA Protein Assay Kit (Thermo).

### Immunoblotting

Protein extracts were electrophoresed on 4% to 12% Bis-Tris gel, transferred to nitrocellulose membranes (Invitrogen), and blocked for 1 hr at room temperature with Blocking One (Nacalai Tesque, Inc., Kyoto, Japan). The membranes were washed three times with 0.1% Tween-20 in Tris-buffered saline and incubated with antibody for GAD1 (Santa Cruz Biotechnology, Dallas, TX) and β-catenin (Novus Biologicals, Littleton, CO) overnight at 4°C and GAPDH (Thermo) for 1 hr at room temperature. The membranes were washed again and incubated with a anti-rabbit or anti-mouse IgG horseradish peroxidase conjugate (Promega, Madison, WI) as a secondary antibody for 1 hr at room temperature. Finally, the membranes were detected using SuperSignal West Pico Chemiluminescent Substrate (Thermo), and immunoblotting was visualized by exposing the membranes to ATTO Light-Capture II (ATTO, Tokyo, Japan). Signal intensities were quantitated using the CS Analyzer version 3.0 software (ATTO).

### IHC

IHC of 4-μm sections of paraffin-embedded specimens was performed using mouse anti-GAD1 monoclonal antibody (Santa Cruz Biotechnology). Briefly, after deparaffinization and hydration, the endogenous peroxidase activity was quenched by a 30-min incubation in a mixture of 0.3% hydrogen peroxide solution in 100% methanol, after which the sections were blocked for 2 hr at room temperature with 1.5% blocking serum (Santa Cruz Biotechnology) in PBS before reaction overnight with anti-GAD1 antibody (1:100 dilution) at 4°C in a moist chamber. Upon incubation with the primary antibody, the specimens were washed three times in PBS and treated with Envision reagent (DAKO, Carpinteria, CA) followed by color development in 3,3′-diaminobenzidine tetrahydrochloride (DAKO). The slides then were lightly counterstained with hematoxylin, dehydrated with ethanol, cleaned with xylene, and mounted. To avoid non-specific binding, an immunizing peptide blocking experiment was performed. As a negative control, triplicate sections were immunostained without exposure to primary antibodies. To quantify the status of the GAD1 protein expression in those components, we used an IHC scoring system to quantitatively evaluate the IHC staining, described previously [22-24]. We counted 300 cells/one field of vision. The staining intensity (0, negative; 1, weak; 2, moderate; 3, intense) and the number of positive cells in the field of vision then were multiplied to calculate the IHC score using the formula: IHC score = 0 × (number of negatively stained cells in the field) + 1 × (number of weakly stained cells in the field) + 2 × (number of moderately stained cells in the field) + 3 × (the highest score for normal tissue). Cases with a GAD1 IHC score exceeding 103 (maximal score within +3 standard deviations of the mean of normal tissues) were defined as GAD1-positive. Two independent pathologists, both masked to the patients’ clinical status, made these judgments.

### Stable transfection of GAD1 shRNA

A total of 2 × 10^5^ OSCC-derived cells (HSC2 and HSC3) were seeded into each well of 6-well plates in DMEM F-12 HAM (Sigma) containing 10% FBS (Sigma) without antibiotics. GAD1 shRNA (shGAD1, Santa Cruz Biotechnology) and the control shRNA (mock, Santa Cruz Biotechnology) vectors were transfected into OSCC-derived cells with Lipofectamine LTX (Invitrogen) and Plus Reagents (Invitrogen). After transfection, the cells were isolated using a culture medium containing 2 μg/mL Puromycin (Invitrogen). After 3 to 4 weeks, resistant cell clones were picked and transferred to 6-well plates and expanded gradually to 10-cm dishes. At 90% confluence, qRT-PCR and immunoblotting were performed to assess the efficiency of GAD1 knockdown.

### 3-Mercaptopropionic acid (3-MPA) treatment

To study the effect of decreased GAD1 activity, we used 3-MPA, a strong competitive inhibitor, at the active GAD1 site [25]. Because several studies has reported that the Ki of 3-MPA ranges from 2.7 to 5.1 μM [26-28], we used 3-MPA (Sigma) at a concentration of 5 μM for functional analyses.

### Cellular growth

To evaluate the effect of GAD1 knockdown on cellular proliferation, we analyzed cellular growth in shGAD1 and mock cells. These transfectants were seeded in 6-well plates at a density of 1 × 10^4^ viable cells/well. The experiments were carried out for 168 hr, and the cells were counted every 24 hr. At the indicated time point, the cells were trypsinized and counted using a hemocytometer in triplicate samples. We also performed a cellular growth assay using 3-MPA-treated cells.

### Invasiveness assay

We evaluated the effect of GAD1 knockdown on cellular invasiveness. A total of 2.5 × 10^5^ cells were seeded on a polyethylene terephthalate membrane insert with a pore size of 3 μm in a transwell apparatus (Becton-Dickinson Labware, Franklin Lakes, NJ). In the lower chamber, 1 ml of DMEM with 10% FBS was added. After the cells were incubated for 48 hr at 37°C, the insert was washed with PBS, and the cells on the top surface of the insert were removed with a cotton swab. Cells adhering to the lower surface of the membrane were fixed with methanol and stained with crystal violet. The numbers of cells invading the pores in five random fields were counted using a light microscope at × 100 magnification. We also performed the invasiveness assay using the 3-MPA-treated cells.

### Migratory assay

shGAD1 and mock cells were seeded in a 6-well plate until they reached full confluence in a monolayer. One wound was created in the middle of each well using a micropipette tip. The plate was incubated at 37°C at 5% CO_2_. The results were visualized by measuring the wound spaces. The mean value was calculated from data obtained from three separate chambers. We also performed a migratory assay using 3-MPA-treated cells.

### Casein zymography

The cells were cultured in serum-free DMEM for 48 hr. The cell culture media were then concentrated using Centrifugal Filter Units (Merck Millipore, Billerica, MA). The concentrated proteins were loaded on precast 12% Novex zymogram blue casein gels (Invitrogen) to measure MMP-7 proteolytic activity. After electrophoresis, the gels were renatured in Novex Zymogram Renaturing Buffer (Invitrogen) for 30 min at room temperature and then incubated at 37°C in Novex Zymogram Developing Buffer (Invitrogen) to allow degradation of the substrate in the gel matrix. Enzymatic activity was visualized as a clear band against a blue background [29-31].

### Statistical analysis

Statistical significance was determined using Fisher’s exact test or the Mann–Whitney U test. *P* < 0.05 was considered significant. The data are expressed as the mean ± standard error of the mean (SEM).

## Results

### Evaluation of GAD1 expression in OSCC-derived cell lines

**Figure 1:**
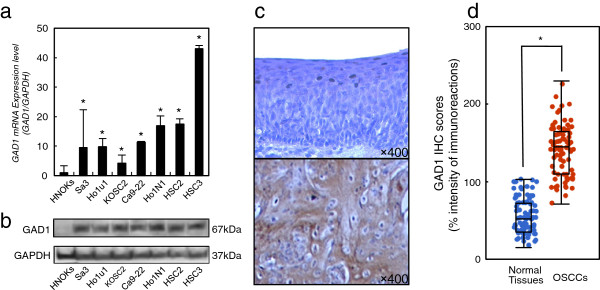
**Evaluation of GAD1 expression in OSCC-derived cell lines. a** Quantification of *GAD1* mRNA levels in OSCC-derived cell lines by qRT-PCR analysis. All OSCC-derived cell lines have significant up-regulation of *GAD1* mRNA compared with that in the HNOKs. Data are expressed as the mean ± SEM of values from three assays (*P < 0.05, Mann–Whitney U test). **b** Immunoblotting analysis of GAD1 protein in the OSCC-derived cell lines and HNOKs. GAD1 protein expressions are up-regulated in all OSCC-derived cell lines examined compared with that in the HNOKs. **c** Evaluation of GAD1 protein expression in primary OSCCs representative IHC results for GAD1 protein in normal tissue and primary OSCC. Original magnification, ×400. Scale bars, 10 μm. Strong GAD1 immunoreactivity is detected in primary OSCCs. Normal oral tissues show almost weak immunostaining. **d** The status of GAD1 protein expression in normal oral tissues and primary OSCCs (n = 80). The GAD1 IHC scores for normal oral tissues and OSCCs range from 15 to 103 (median, 52) and 71 to 230 (median, 145), respectively. GAD1 protein expression levels in OSCCs are significantly (*P < 0.001, Mann–Whitney U test) higher than in normal oral tissues.

We performed qRT-PCR and immunoblotting using OSCC-derived cell lines (Sa3, HO-1-u-1, KOSC-2, Ca9-22, HO-1-N-1, HSC-2, and HSC-3) and HNOKs (Figure 1a, b). *GAD1* mRNA was significantly (*P* < 0.05) up-regulated in all OSCC-derived cell lines compared with the HNOKs. Figure 1b shows representative results of immunoblotting analysis of GAD1 (67 kDa). All OSCC-derived cell lines had a significant (*P* < 0.05) increase in GAD1 protein expression compared with the HNOKs. Expression analyses indicated that both transcription and translation products of this molecule were highly expressed in OSCC-derived cell lines.

### Evaluation of GAD1 expression in primary OSCCs

We analyzed the GAD1 protein expression in primary OSCCs and paired normal oral tissues from 80 patients using the IHC scoring system. Figure 1c shows representative IHC results for GAD1 protein in normal oral tissues and primary OSCCs. Strong GAD1 immunoreactions were detected in the cytoplasm in the OSCCs. The GAD1 IHC scores for normal oral tissues and OSCCs ranged from 15 to 103 (median, 52) and 71 to 230 (median, 145), respectively. The GAD1 IHC score in primary OSCCs was significantly (*P* < 0.001) higher than in normal oral tissues (Figure 1d).

### Establishment of GAD1 knockdown cells

**Figure 2:**
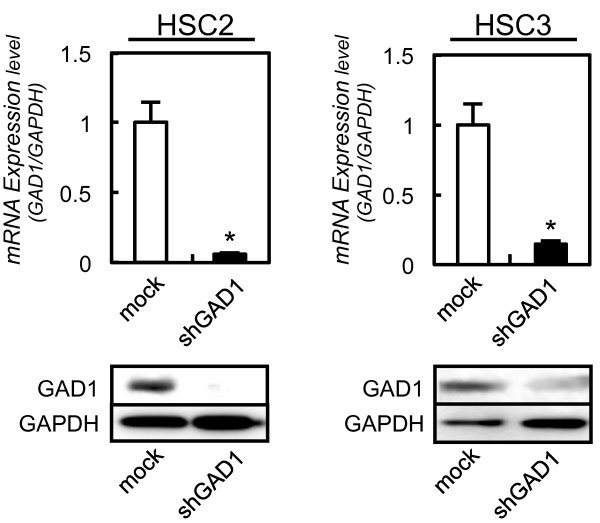
**Expression of GAD1 in GAD1 knockdown cells.** qRT-PCR shows that *GAD1* mRNA expression in the shGAD1 cells (HSC2 and HSC3-derived transfectants; 2 clones each) are significantly lower than that in the mock cells (*P < 0.05, Mann–Whitney U test). Immunoblotting analysis shows that the GAD1 protein levels in shGAD1 cells (HSC2 and HSC3-derivrd transfectants; 2 clones each) have decreased markedly compared with that in mock cells.

To assess the GAD1 functions in oral cancer, shRNA transfection was carried out in the OSCC-derived cells (HSC2 and HSC3). Expressions of GAD1 mRNA and protein in shGAD1 cells were significantly (*P* < 0.05) lower than in mock cells (Figure 2).

### Functional analyses of GAD1 knockdown cells

**Figure 3:**
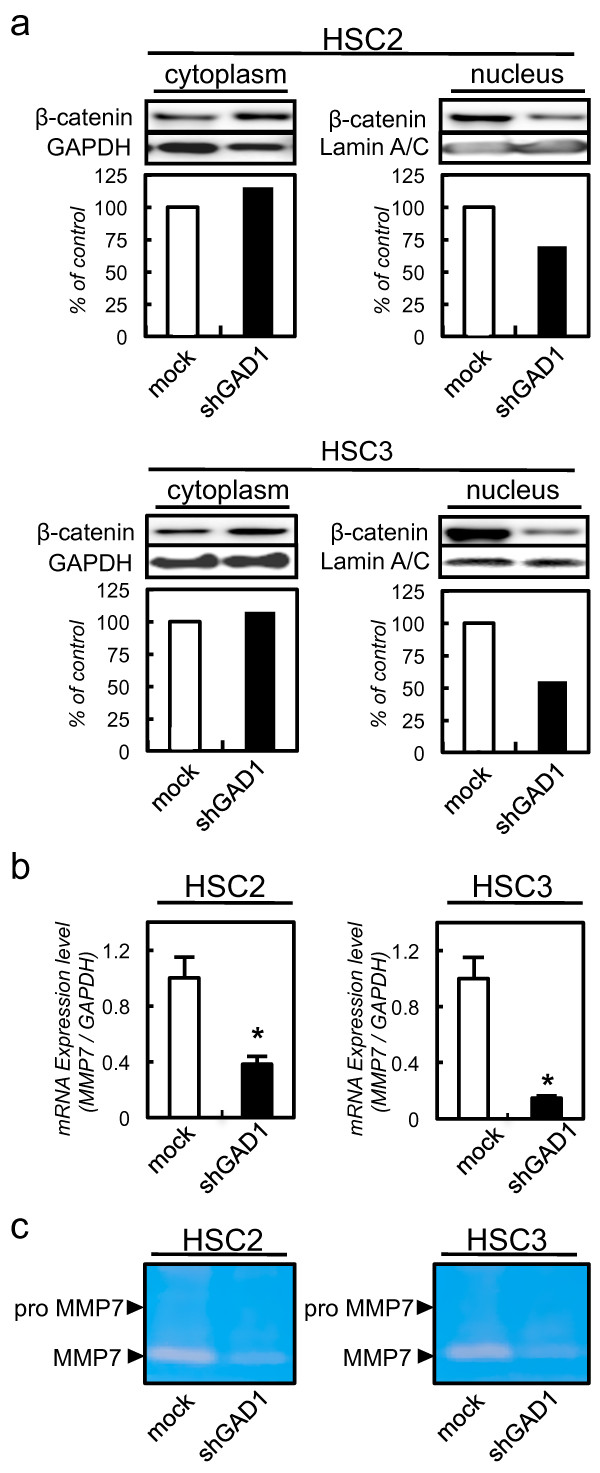
**GAD1 knockdown cells suppress translocation of β-catenin to the nucleus and MMP7 activation. a** Immunoblotting analysis of β-catenin in the nucleus of shGAD1 cells. The expression of β-catenin in the nucleus of shGAD1 cells has decreased markedly compared with that in mock cells. β-catenin expression in the cytoplasm does not differ significantly between shGAD1 cells and mock cells. **b** Quantification of *MMP7* mRNA levels in shGAD1 cells by qRT-PCR analysis. *MMP7* mRNA is significantly down-regulated in shGAD1 cells compared with mock cells. **c** Casein zymography analysis of MMP7 activity in shGAD1. Cell culture media are collected and concentrated, MMP7 activity is analyzed by casein zymography. MMP7 secretion is decreased significantly in shGAD1 cells compared with mock cells.

β-catenin, which is located along the cell membrane and cytoplasm in normal epithelial cells, is involved in cellular adhesion and migration [32]. In cancer epithelial cells, β-catenin is translocated into the nucleus, which activates oncogenes including MMP-7 [33]. To assess the translocation of β-catenin in shGAD1 cells, we performed immunoblotting analysis using shGAD1 and mock cells. The expression of β-catenin in the nucleus was suppressed in shGAD1 cells compared with mock cells. The expressions of β-catenin in the cytoplasm did not differ significantly between the shGAD1 and mock cells (Figure 3a). To evaluate the *MMP7* mRNA expression, we also performed qRT-PCR using shGAD1 and mock cells. The expression of *MMP7* mRNA decreased significantly in shGAD1 cells compared with mock cells (Figure 3b). Using casein zymography, we also detected secreted MMP7 in shGAD1 and mock cells. The MMP7 secretion was suppressed significantly (*P* < 0.05) in shGAD1 cells compared with mock cells (Figure 3c).

**Figure 4:**
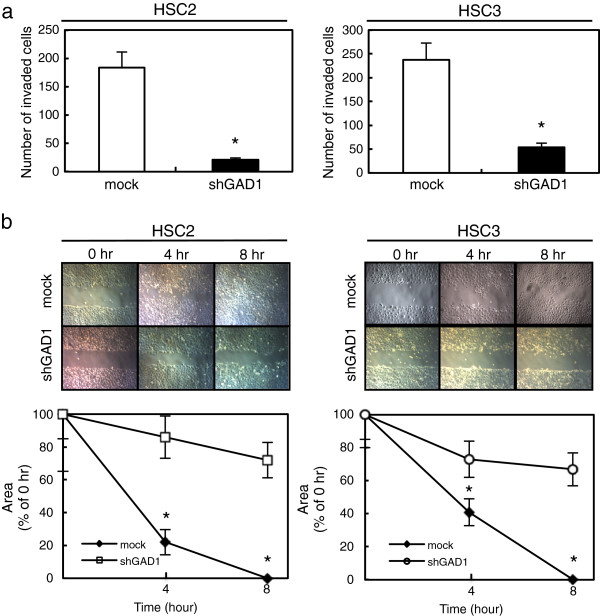
**Functional analyses of GAD1 knockdown cells. a** Invasiveness assay of the shGAD1 cells. After crystal violet staining, the numbers of cells invading the pores is counted (×100 magnification). The numbers penetrating the shGAD1 cells are significantly (*P < 0.05, Mann–Whitney U test) greater compared with mock cells. Scale bars, 100 μm. **b** Migratory assay of shGAD1 cells. The wound area has decreased significantly (*P < 0.05, Mann–Whitney U test) in the culture of mock cells after 8 hr, whereas there is still a gap in the shGAD1 cells. Original magnification, ×100. Scale bars, 100 μm.

We also performed cellular proliferation, invasiveness, and migratory assays to evaluate the biologic effects of shGAD1 cells. A cellular proliferation assay showed similar growth curves for shGAD1 and mock cells, indicating that down-regulation of GAD1 did not affect cellular proliferation (data not shown). The invasiveness assay showed that the number of penetrating shGAD1 cells decreased compared with mock cells (Figure 4a). The migratory assay showed that the wounds in the shGAD1 cells closed later than in the mock cells when we visually monitored the area of uniform wounds in confluent cell cultures (Figure 4b).

### Functional analyses of 3-MPA-treated cells

**Figure 5:**
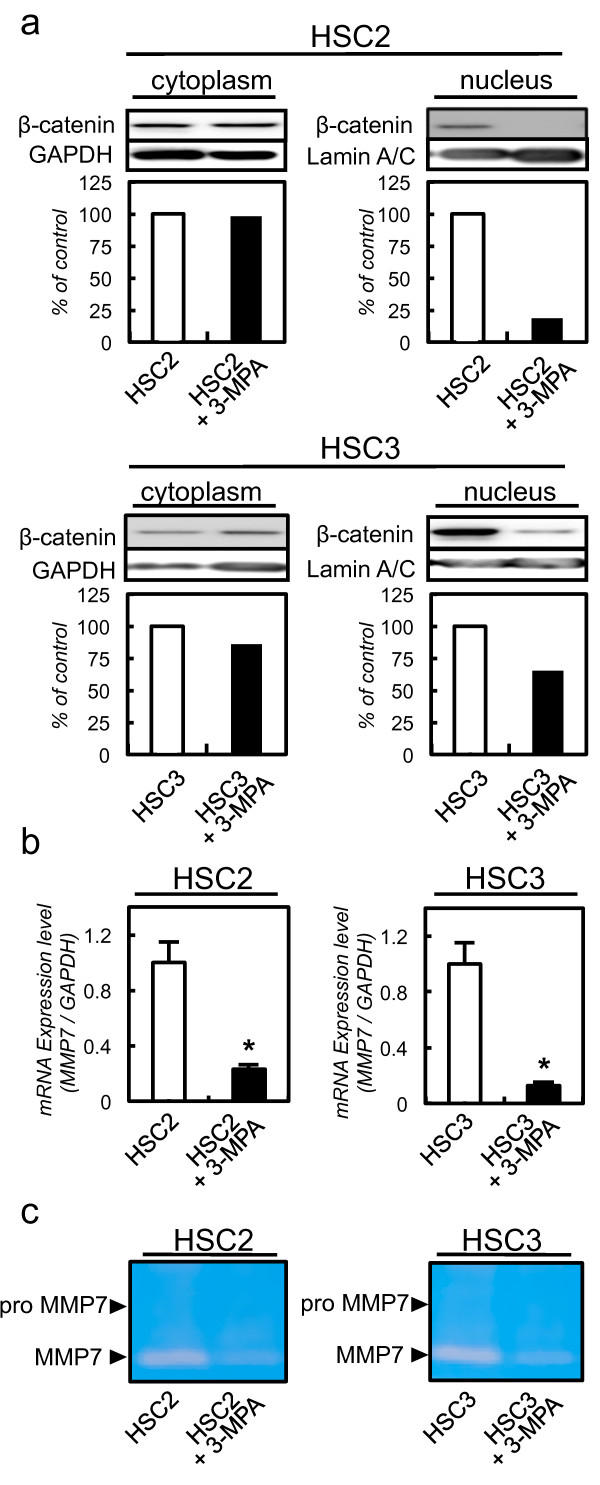
**3-MPA-treated cells suppress translocation of β-catenin to the nucleus and MMP7 activation. a** Immunoblotting analysis of β-catenin in the nuclei of 3-MPA-treated cells. β-catenin expression in the nucleus of 3-MPA-treated cells is decreased markedly compared with that in control cells. β-catenin expression in the cytoplasm does not differ significantly between 3-MPA-treated cells and control cells. **b** Quantification of *MMP7* mRNA level in 3-MPA-treated cells by qRT-PCR analysis. *MMP7* mRNA is significantly down-regulated in 3-MPA-treated cells compared with control cells. **c** Casein zymography analysis of MMP7 activity in 3-MPA-treated cells. Cell culture media are collected and concentrated, MMP7 activity is analyzed by casein zymography. MMP7 secretion is decreased significantly in 3-MPA-treated cells compared with control cells.

We also performed functional analysis using 3-MPA. To assess the translocation of β-catenin in 3-MPA-treated cells, we performed immunoblotting analysis using 3-MPA-treated and control cells. The expression of β-catenin in the nucleus was suppressed in 3-MPA-treated cells. The expression of β-catenin in the cytoplasm did not differ significantly between the 3-MPA-treated cells and control cells (Figure 5a). To evaluate the *MMP7* mRNA expression, we also performed qRT-PCR using 3-MPA-treated and control cells. The *MMP7* mRNA expression decreased significantly in the 3-MPA-treated cells compared with control cells (Figure 5b). We also detected MMP7 secreted by casein zymography in 3-MPA and control cells. The secretion of MMP7 was suppressed in 3-MPA-treated cells compared with control cells (Figure 5c).

**Figure 6:**
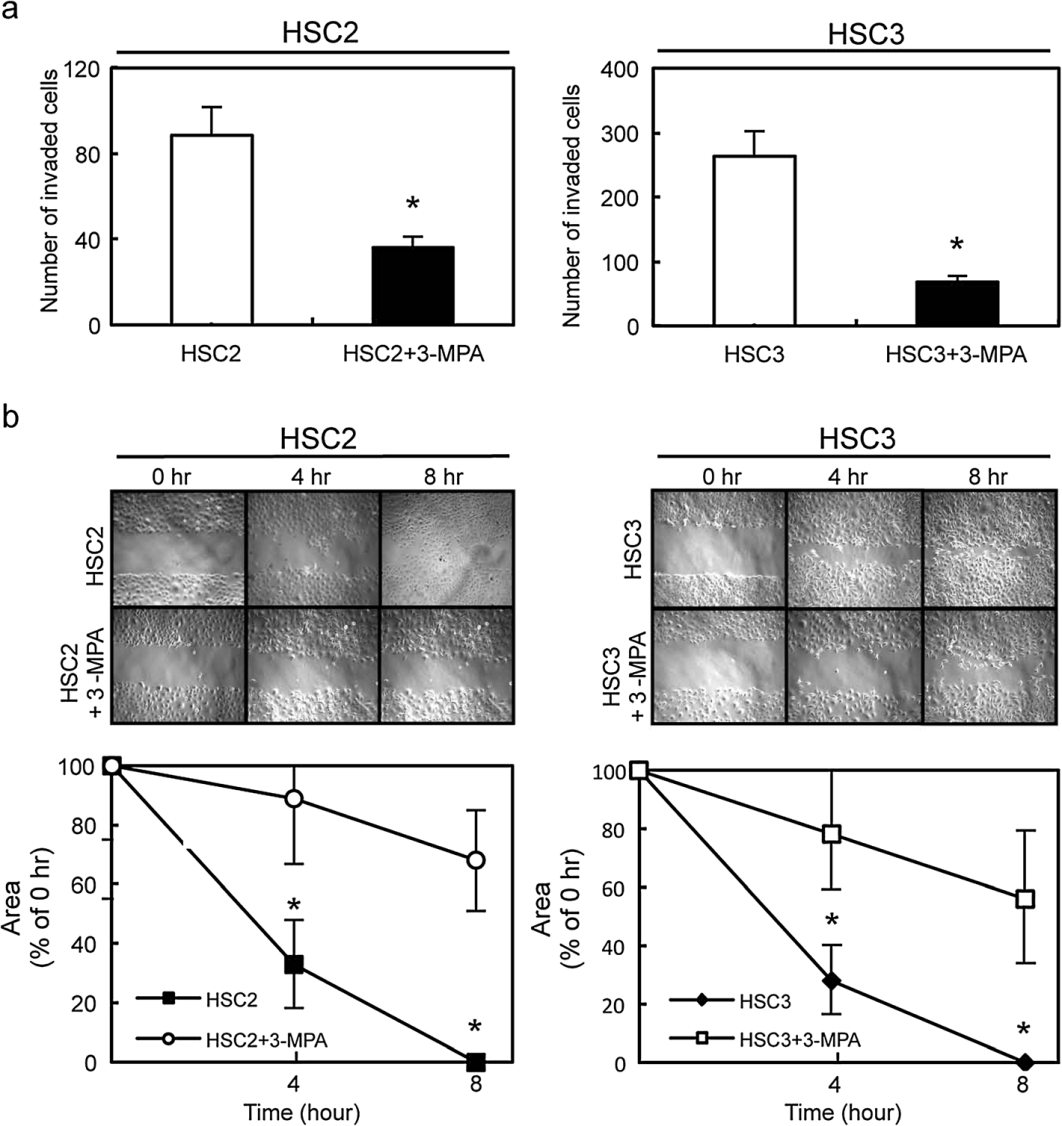
**Functional analysis of the 3-MPA-treated cells. a** Invasiveness assay of the 3-MPA-treated cells. After crystal violet staining, the numbers of cells invading the pores are counted (×100 magnification). The numbers penetrating the 3-MPA-treated cells are significantly (*P < 0.05, Mann– Whitney U test) greater compared with control cells. Scale bars, 100 μm. **b** Migratory assay of 3-MPA-treated cells. The wound area is decreased significantly (*P < 0.05, Mann–Whitney U test) in the culture of control cells after 8 hr, whereas there is still a gap in the 3-MPA-treated cells. Original magnification, ×100. Scale bars, 100 μm.

We performed cellular proliferation, invasiveness, and migratory assays to evaluate the biologic effects of 3-MPA-treated cells. The cellular proliferation assay showed similar growth curves for 3-MPA-treated and control cells, indicating that inhibition of GAD1 did not affect cellular proliferation (data not shown). The invasiveness assay showed that the number of penetrating 3-MPA-treated cells decreased compared with control cells (Figure 6a). The migratory assay showed that the wounds in the 3-MPA-treated cells closed later than in control cells (Figure 6b) when we visually monitored the area of uniform wounds in confluent cell cultures.

### Expression of GAD1 and clinicopathological variables of primary OSCCs

Table 1 shows the correlations between the clinicopathologic characteristics of patients with OSCC and the status of the GAD1 protein expression using the IHC scoring system. Among the clinical classifications, GAD1-positive OSCCs were significantly (*P* = 0.011) correlated with regional lymph node metastasis.

**Table 1:** Correlation between GAD1 expression and clinical classification in OSCC

**Clinical classification**		**Total**	**Result of immunostaining**		***P *** **value**
			**No. of patients (%)**		
			**GAD1 (high)**	**GAD1 (low)**	
Age at surgery					
	< 60	17	15 (88%)	2 (12%)	0.527
	≧ 60	63	52 (83%)	11 (17%)	
Gender					
	Male	34	29 (85%)	5 (15%)	0.401
	Female	46	38 (83%)	8 (17%)	
T-primary tumor size					
	T1	8	6 (75%)	2 (25%)	0.263
	T2	48	38 (79%)	10 (21%)	
	T3	14	13 (93%)	1 (7%)	
	T4	10	10 (100%)	0 (0%)	
N-regional lymph node					
	N (negative)	48	36 (75%)	12 (25%)	0.011*
	N (positive)	32	31 (97%)	1 (3%)	
Stage					
	I	8	6 (75%)	2 (25%)	0.075
	II	40	30 (75%)	10 (25%)	
	III	20	19 (95%)	1 (5%)	
	IV	12	12 (100%)	0 (0%)	
Histopathlogical type					
	Well	54	46 (85%)	8 (15%)	0.441
	Moderately	16	14 (88%)	2 (12%)	
	Poorly	10	7 (70%)	3 (30%)	
Tumor site					
	Gingiva	20	18 (90%)	2 (10%)	0.243
	Tongue	52	44 (85%)	8 (15%)	
	Buccal mucosa	4	2 (50%)	2 (50%)	
	Oral floor	4	3 (75%)	1 (25%)	

GAD1(high), up-regulated GAD1; GAD1 (low), down-regulated GAD1; ^*^P < 0.05.

## Discussion

GAD1 was overexpressed in OSCC-derived cell lines and new functions of GAD1 were related closely to cellular invasiveness and migration in oral cancer. GAD1 knockdown and 3-MPA-treated cells had suppressed β-catenin levels in the nucleus and secretion of MMP7. Surprisingly, GAD1-positive OSCCs were significantly (*P* < 0.05) associated with regional lymph node metastasis (Table 1).

GAD isoforms, GAD1 and GAD2, are derived from a common ancestral gene [34]. GAD2 is localized to the nerve terminal and is reversibly bound to the membrane of synaptic vesicles, which has been linked with lower birth weights and additional risk for metabolic diseases [35], whereas GAD1 is a cytosolic enzyme distributed throughout the organs and central nervous system [36]. The enzymatic functions of GAD1 and GAD2 are almost similar; however, their functions remain unclear in cancer tissues [37]. Since our previous microarray data showed that GAD1 is up-regulated significantly in OSCCs [38], we focused on GAD1 in the current study.

β-catenin plays crucial and diverse roles in cadherin-mediated cell-cell adhesion, Wnt signal transduction, gene activation, and tumoral formation [39-41]. Although the interaction mechanism between GAD1 and β-catenin has not yet been reported, the current data suggested that GAD1 expression controls β-catenin localization. β-catenin in nuclei binds to the TCF/LEF in several types of cancers for transcriptional activation of downstream genes, such as *MMP7*, *cyclinD1*, and *c-myc*[42-46], which play important roles in carcinogenesis and metastasis.

We then investigated MMP7 secretion, a downstream candidate of GAD1/β-catenin interaction, because MMP7 often is overexpressed in human cancer tissues and associated with cancer cell invasiveness by proteolytic cleavage of the ECM substrates and degradation of basement membrane proteins [47-50]. Interestingly, we found that GAD1 knockdown and 3-MPA-treated cells inhibited MMP7 secretion by decreasing nuclear translocation of β-catenin. We speculated that the GAD1/β-catenin/MMP7 interaction affects cancer cell behaviors, such as cellular invasiveness and migration. In addition to the *in vitro* data that down-regulation of GAD1 led to low cellular invasiveness and migratory abilities, patients with GAD1-negative OSCC had a low risk of regional lymph node metastasis. Consistent with our hypothesis, the GAD1/β-catenin/MMP7 interaction is correlated closely with metastasis both *in vitro* and *in vivo*.

## Conclusion

Our results showed that oral cancer carcinogenesis overexpression of GAD1 occurs frequently and that it might be closely associated with invasion and metastasis of OSCC by β-catenin translocation and MMP7 activation, while further studies are needed to research the GAD1/β-catenin/MMP7 interaction, the current data indicated that GAD1 is likely a molecular marker for early detection of lymph node metastasis and an efficacious treatment target for preventing cancer metastasis in OSCCs.

## Abbreviations

GAD1: Glutamate acid decarboxylase 1); GABA: Gamma-aminobutyric acid; MMP7: Matrix metalloproteinase-7; 3-MPA: 3-mercaptopropionic acid.

## Competing interests

The authors declare that they have no competing interests.

## Authors’ contributions

Conceived and designed the experiments: RK, AK, HT, UK. Performed the experiments: RK, AK, UK. Analyzed the data: RK, AK, UK. Contributed reagents/materials/analysis tools: RK, AK, TK, CF, YK, MH, YE-S, KO, MS, UK, HT. Wrote the paper: RK, AK, UK, HT. All authors read and approved the final manuscript.

## Pre-publication history

The pre-publication history for this paper can be accessed here:

http://www.biomedcentral.com/1471-2407/13/555/prepub
